# High-risk oral leukoplakia is associated with aberrant promoter methylation of multiple genes

**DOI:** 10.1186/s12885-016-2371-5

**Published:** 2016-06-03

**Authors:** Masanobu Abe, Satoshi Yamashita, Yoshiyuki Mori, Takahiro Abe, Hideto Saijo, Kazuto Hoshi, Toshikazu Ushijima, Tsuyoshi Takato

**Affiliations:** Department of Oral & Maxillofacial Surgery, University of Tokyo Hospital, 7-3-1 Hongo, Bunkyo-ku, Tokyo, 113-8655 Japan; Division for Health Service Promotion, University of Tokyo, Tokyo, Japan; Division of Epigenomics, National Cancer Center Research Institute, Tokyo, Japan; Department of Dentistry, Oral & Maxillofacial Surgery, Jichi Medical University, Tochigi, Japan

**Keywords:** Methylation, Promoter methylation, Gene silencing, Oral squamous cell carcinoma, Oral leukoplakia

## Abstract

**Background:**

Early detection of oral squamous cell carcinomas (OSCCs) is urgently needed to improve the prognosis and quality of life (QOL) of patients. Oral leukoplakias (OLs), known as the most common premalignant lesions in the oral cavity, often precede OSCCs. Especially, OLs with dysplasia are known to have a high risk of malignant transformation. Here, we searched for the promoter methylation characteristic of high-risk OLs.

**Methods:**

To identify methylation-silenced genes, a combined analysis of methylated DNA immunoprecipitation (MeDIP) − CpG island (CGI) microarray analysis and expression microarray analysis after treatment with a demethylating agent was performed in two OSCC cell lines (Ca9–22 and HSC-2). The methylation statuses of each gene were examined by methylation-specific PCR.

**Results:**

A total of 52 genes were identified as candidates for methylation-silenced genes in Ca9-22 or HSC-2. The promoter regions of 13 genes among the 15 genes randomly selected for further analysis were confirmed to be methylated in one or more of five cell lines. In OSCC tissues (*n* = 26), 8 of the 13 genes, *TSPYL5*, *EGFLAM, CLDN11, NKX2-3, RBP4, CMTM3, TRPC4*, and *MAP6,* were methylated. In OL tissues (*n* = 24), seven of the eight genes*,* except for *EGFLAM,* were found to be methylated in their promoter regions. There were significantly greater numbers of methylated genes in OLs with dysplasia than in those without dysplasia (*p* < 0.0001).

**Conclusions:**

OLs at high risk for malignant transformation were associated with aberrant promoter methylation of multiple genes.

**Electronic supplementary material:**

The online version of this article (doi:10.1186/s12885-016-2371-5) contains supplementary material, which is available to authorized users.

## Background

Oral cancer is a major public health problem worldwide, and OSCC is the most common type of oral cancer. The survival rates of patients with OSCCs have remained largely unchanged for decades, with a 5-year survival rate of around 50 % despite advances in therapeutics [1–4]. In addition to that, even when patients with advanced OSCCs survive after surgery, large tissue defects of the maxillofacial region pose a serious problem. Therefore, early and accurate detection of OSCCs is important not only to improve the survival rate of patients with OSCCs but also to maintain good QOL of the patients.

For the early detection of OSCCs, a finding of oral premalignant lesions with high-risk malignant transformation is important. OL is the most common premalignant lesion in the oral cavity, and OLs often precede OSCCs. The transition frequency from OLs into OSCCs ranges widely, from 0.13 % to 36.4 % [5]. Histologically, the presence of dysplasia is often associated with the development of OSCCs [6–8]. However, the molecular mechanism underlying malignant transformation of OLs has not been elucidated yet, and molecular markers to identify patients at higher risk of developing OSCC have not been isolated [9].

As a molecular marker to identify lesions with a higher risk of malignant transformation, DNA methylation might be useful [10–14]. The accumulation of aberrant methylation in non-cancerous lesions, such as gastric mucosae with *Helicobacter pylori* infection, produces epigenetic field defects leading to malignant transformation [15, 16]. In the field of oral malignancy, although many reports describe methylation silencing in OSCCs [17–21], few reports focus on methylation in OLs, especially OLs with a high-risk of malignant transformation [18, 22–26].

In this study, we aimed to identify aberrant promoter methylation in OLs at high risk of malignant transformation.

## Methods

### Cell lines, tissue samples, and DNA extraction

Human OSCC cell lines (Ca9–22, HSC-2, HO-1-N-1, HSC-3 and SCC-4) were purchased from the Human Science Research Resources Bank (HSRRB, Osaka, Japan). A total of 24 OL tissues (average age, 64.0 years [range, 38–84 years]; 10 male and 14 female) and a total of 26 OSCC tissues (average age, 64.6 years [range, 42–89 years]; 17 male and 9 female) were obtained from patients who underwent biopsies or operations at the University of Tokyo Hospital between Dec. 2009 and Nov. 2011. The OSCCs were graded according to the Union for International Cancer Control (UICC)’s TNM classification. OL was defined as “a predominantly white lesion of the oral mucosa that can not be characterized as any other definable lesion”[27]. The presence or absence of dysplasia in OLs is determined by the degree of cellular abnormality above the epithelial basement membrane as originally defined by the World Health Organisation (WHO) [28]. Normal oral mucosae were obtained from 16 healthy volunteers. Samples were stored in RNAlater (Applied Biosystems, Foster City, CA, USA) at -80 °C until the extraction of genomic DNA. Genomic DNA was extracted by the phenol-chloroform method. This research was approved by the research ethics committee of Graduate School of Medicine and Faculty of Medicine, The University of Tokyo, approval #2819-(1), and informed consent was obtained from all patients and volunteers. Each patient’s tobacco smoking history was obtained in an interview.

### 5-Aza-2′-deoxycytidine treatment

Ca9–22 and HSC-2 cells were seeded at a density of 2 × 10^5^ cells ⁄ 10 cm plate on day 0. For 5-aza-2′-deoxycytidine (5-aza-dC; Sigma, St Louis, MO, USA) treatment, the cells were exposed to medium containing 3-μM 5-aza-dC or control medium for 24 h on days 1 and 3, and then harvested on day 5. The doses of 5-aza-dC were adjusted so that the growth of the treated cells was suppressed to 40–80 % that of nontreated cells.

### Methylated DNA immunoprecipitation (MeDIP) − CpG island (CGI) microarray analysis

MeDIP − CGI microarray analysis was performed as previously described [29, 30]. Briefly, 5 μg of genomic DNA was immunoprecipitated with an anti-5-methylcytidine antibody (Diagnode, Liége, Belgium), and the precipitated DNA and input DNA were labeled with Cy5 and Cy3, respectively. A human CGI oligonucleotide microarray (Agilent Technologies, Santa Clara, CA, USA) was hybridized with the labeled probes and scanned with a G2565BA microarray scanner (Agilent Technologies). Scanned data were processed with Feature Extraction 9.1 and ChIP Analytics 1.3 software (Agilent Technologies). The signal of the probe was converted into a “Me value,” which represents the methylation level as a value from 0 (unmethylated) to 1 (methylated) [29]. Differentially methylated regions were detected by a comparison of the Me values of the two samples. When three or more consecutive probes in a locus showed differences in the Me value larger than 0.6, the locus was considered to have different methylation statuses. Promoter regions of three genes (*HOXA11*, *NPY*, and *UCHL1*) reported as frequently methylated in multiple cancers, including OSCCs, were used as a methylated control [31–33]. Promoter regions of three genes (*ACTB*, *B2M,* and *GAPDH*) known as housekeeping genes were used as unmethylated control.

### Gene expression analysis by oligonucleotide microarray

Expression microarray analysis was performed by a GeneChip Human Genome U133 Plus 2.0 expression microarray (Affymetrix, Santa Clara, CA, USA). From 8 μg of total RNA, first-strand cDNA was synthesized with SuperScript III reverse transcriptase (Invitrogen) and T7-(dT)24 primer (Amersham Biosciences, Little Chalfont, UK). Double-stranded cDNA was then synthesized, and biotin-labeled cRNA was synthesized using a Bio-Array HighYield RNA transcript-labeling kit (Enzo Life Sciences, Farmingdale, NY, USA). Twenty micrograms of labeled cRNA was fragmented and hybridized to the GeneChip oligonucleotide microarray with a GeneChip hybridization control kit. The microarray was stained and scanned according to the Affymetrix protocol. The scanned data were processed using GeneChip operating software 1.4. The signal intensity of each probe was normalized so that the average signal intensity of all the probes on a microarray would be 500. The average signal intensity of all the probes for a gene was used as its transcription level. Genes were classified into those with high (>1000), moderate (250–1000), or low (<250) transcriptions according to their signal intensities [30].

### Sodium bisulfite modification and methylation-specific PCR (MSP)

Sodium bisulfite treatment was performed as described previously [29] using 500 ng of DNA digested with *Bam*HI (Toyobo, Tokyo, Japan) and suspended in 20 μl of Tris-EDTA (TE) buffer. For MSP, 1 μl of solution was used for PCR reaction with primers specific to methylated (Additional file 1: Table S1) and with primers that targeted the *Alu* repeat sequence; the latter were used as a control of the amount of bisulfite-treated DNA [34]. Fully methylated DNA was prepared by methylating genomic DNA using *Sss*I-methylase (New England Biolabs, Beverly, MA, USA). Fully unmethylated DNA was prepared by amplifying genomic DNA with phi29 DNA polymerase (GenomiPhi DNA Amplification kit; GE Healthcare UK, Buckinghamshire, UK).

### Statistical analysis

Associations between methylation status and various clinical parameters were evaluated by Fisher’s exact test (two-sided). SPSS Statistics (IBM Corporation, Somers, NY, USA) software version 21.0 (SPSS Inc., Chicago, IL, USA) was used for analysis. P values < 0.05 were considered to indicate significance.

## Results

### Chemical genomic screening of methylation-silenced genes in OSCC cell lines

**Fig. 1:**
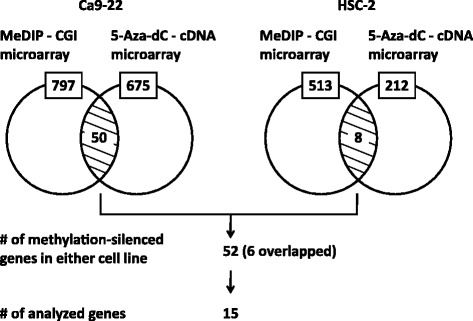
Isolation of methylation-silenced genes in two OSCC cell lines. MeDIP–CGI microarray analysis showed that 797 and 513 promoter CGIs were hypermethylated in Ca9–22 and HSC-2, respectively. 5-aza-dC – cDNA microarray analysis showed that the expression levels of 675 and 212 genes were upregulated in Ca9–22 and HSC-2, respectively. By integrating these data, 50 and 8 genes were indicated methylation-silenced in Ca9–22 and HSC-2, respectively. After duplicates were removed, 52 genes were indicated to be methylation-silenced in either cell line. Of these 52 genes, 15 genes were randomly selected for further analysis

To identify methylation-silenced genes in OSCC, we took a combined approach of MeDIP–CGI microarray analysis in two OSCC cell lines (Ca9–22, HSC-2) and expression microarray analysis before and after treatment of the two cell lines with a demethylating agent, 5-aza-dC. In the Ca9–22 cell line, the MeDIP − CGI microarray showed that 797 promoter CGIs were hypermethylated, and the expression microarray data showed that the expression levels of 675 genes were upregulated three-fold or more after 5-aza-dC treatment. By integrating the MeDIP − CGI microarray data and the expression microarray data, 50 genes were indicated to be methylation-silenced in the Ca9–22 cell line (Fig. 1). In the HSC-2 cell line, the MeDIP − CGI microarray showed that 513 promoter CGIs were hypermethylated, and the expression microarray data showed that the expression levels of 212 genes were upregulated three-fold or more after 5-aza-dC treatment. By integrating the MeDIP − CGI microarray data and the expression microarray data, eight genes were indicated to be methylation-silenced in the HSC-2 cell line (Fig. 1). After duplicates were removed, 52 genes were indicated to be methylation-silenced in either OSCC cell line. Of these 52 genes, 15 genes were “randomly” selected for further analysis (Fig. 1).

### Methylation profiles of the 15 promoter CGIs in OSCC cell lines

**Fig. 2:**
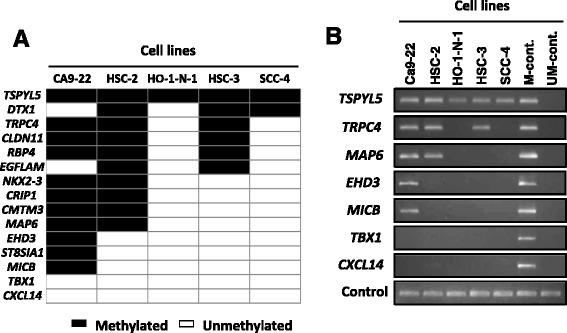
Methylation profiles of 15 promoter CGIs in five OSCC cell lines. **a** Promoter CGIs of 13 of the 15 genes were methylated in one or more of the five OSCC cell lines (Ca9–22, HSC-2, HO-1-N-1, HSC-3 and SCC-4). Closed box, methylated DNA detected; open box, methylated DNA not detected. **b** Representative results of MSP in the five OSCC cell lines are shown. Methylated DNA-specific primer sets were used to detect aberrant DNA methylation. Primer sets that target the *Alu* repeat sequence were used as a control of the amount of bisulfite-treated DNA. M-cont and UM-cont are fully methylated and fully unmethylated DNA, respectively

The selected 15 genes were analyzed in five OSCC cell lines by MSP. Of the 15 genes, 13 were methylated in one or more of these cell lines (Fig. 2a) and selected for further analysis. Representatively, a promoter CGI of *TSPYL5* (*Testis-specific protein, Y-encoded-like 5*) was methylated in all five cell lines (Fig. 2b). A promoter CGI of *TRPC4* (*Transient receptor potential cation channel, subfamily C, member 4*) was methylated in three cell lines.

### Methylation profile of the 13 promoter CGIs in OSCC tissues

**Fig. 3:**
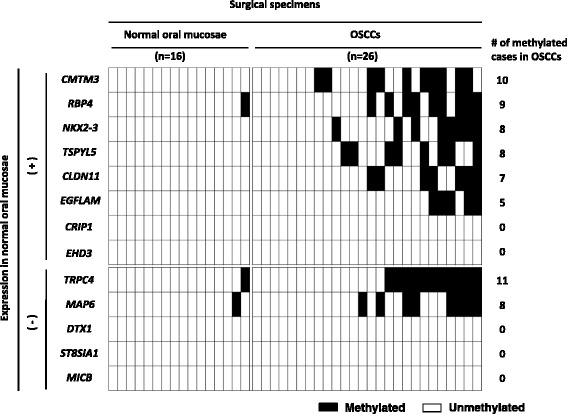
Methylation profiles of the 13 promoter CGIs in OSCC tissues. The methylation statuses of the 13 genes showing promoter methylation in the OSCC cell lines were examined in 16 normal oral mucosae and 26 OSCC tissues. Eight genes were expressed in normal oral mucosae, six of which showed promoter methylation in the OSCC tissues. Five genes were unexpressed in normal oral mucosae, two of which were methylated in their promoter regions in the OSCC tissues. Closed box, methylated DNA detected; open box, methylated DNA not detected

Promoter methylation of genes expressed in normal oral mucosae can affect gene function, and is potentially important for malignant transformation. On the other hand, promoter methylation of genes unexpressed in normal oral mucosae is considered to be passenger methylation during OSCC carcinogenesis [34]. Therefore, the expression levels of the 13 methylated genes in OSCC cell lines were investigated in normal oral mucosae using the GEO database (GEO database; http://www.ncbi.nlm.nih.gov/geo/). Eight of those genes − *CMTM3* (*CKLF-like MARVEL transmembrane domain containing 3*)*, RBP4* (*Retinol-binding protein 4*)*, NKX2–3* (*NK2 homeobox 3*)*, TSPYL5*, *CLDN11* (*Claudin 11*), *EGFLAM* (*EGF-like, fibronectin type III and laminin G domain*), *CRIP1* (*Cysteine-rich protein 1)*, and *EHD3 (EH-domain containing 3)* − were expressed in normal oral mucosae (GSM447398, GSM447399, GSM447408, GSM447404, GSM447406 and GSM447407 in the GEO database). Five of the 13 genes − *TRPC4, MAP6* (*Microtubule-associated protein 6*), *DTX1* (*Deltex homolog 1*)*, ST8SIA1* (*ST8 alpha-N-acetyl-neuraminide alpha-2, 8-sialyltransferase 1*), and *MICB* (*MHC class I polypeptide-related sequence B*) − were unexpressed in normal oral mucosae (Fig. 3).

Of the eight expressed genes, six (*CMTM3*, *RBP4*, *NKX2–3, TSPYL5, CLDN11*, and *EGFLAM*) showed promoter methylation in 10, 9, 8, 8, 7, and 5 of 26 OSCC tissues, respectively. Two of the five unexpressed genes (*TRPC4* and *MAP6*) showed promoter methylation in 11 and 8 of the 26 OSCC tissues, respectively (Fig. 3).

Clinicopathological analysis was performed to examine the association between the methylation status of the eight identified genes and the clinical parameters. The categorizations of cases for each parameter are shown in Additional file 2: Table S2. The methylation status of *TSPYL5* was inversely associated with the differentiation levels of OSCCs (*p* < 0.01) and was prone to be methylated in the tongue rather than in the other sites (*p* < 0.05). However, none of the other genes showed an association between methylation status and age (above average or below average), sex (male or female), stage (early or advanced), or smoking history (presence or absence).

### Methylation profile of the eight promoter CGIs in OL tissues and identification of aberrant methylation in OLs with dysplasia

**Fig. 4:**
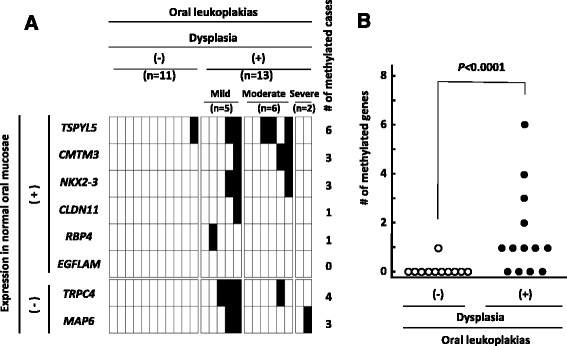
Methylation profiles of the eight promoter CGIs in OL tissues and identification of aberrant methylation in OLs with dysplasia. **a** The methylation statuses of the eight genes showing promoter methylation in OSCC tissues were examined in 24 OL tissues. Six and two of the eight genes were expressed and unexpressed, respectively, in normal oral mucosae. Thirteen and 11 cases were OLs with and without dysplasia, respectively. The OLs with dysplasia were categorized into mild (*n* = 5), moderate (*n* = 6), and severe grade (*n* = 2). Five of the six expressed genes and both of the unexpressed genes showed promoter methylation in OL(s) with dysplasia. **b** When the number of methylated genes was summed and compared between OLs with and without dysplasia, the methylation status showed a significant association with the presence of dysplasia (*p* < 0.0001)

The methylation statuses of the eight (six expressed and two unexpressed) genes that showed promoter methylation in OSCC tissues were examined in 13 and 11 OLs with- and without dysplasia, respectively (Fig. 4a). Of the six expressed genes, five (*CMTM3, RBP4, NKX2–3, TSPYL5,* and *CLDN11*) were methylated in 6, 3, 3, 1, and 1 OL(s), respectively. The two unexpressed genes (*TRPC4* and *MAP6*) were methylated in four and three OLs, respectively.

When the number of methylated genes in an OL was compared between OLs with and without dysplasia, it was found that OLs with dysplasia had significantly more methylated genes (Fig. 4b, *p* < 0.0001). Only one OL tissue without dysplasia, diagnosed as an acanthosis, showed an aberrant promoter methylation. As for individual genes, *TRPC4* methylation was associated with the presence of dysplasia in OLs (*p* = 0.04). The OLs with dysplasia were categorized into mild (*n* = 5), moderate (*n* = 6), and severe grade (*n* = 2), however any association between the grade of dysplasia and methylation status of the identified genes was not observed (Fig. 4a).

Clinicopathological analysis was performed to examine the association between the methylation status of each of the seven genes and the clinical parameters. The categorizations of cases for each parameter are shown in Additional file 2: Table S2. None of the genes showed an association with age, sex, stage, site, or smoking history.

## Discussion

We identified seven genes aberrantly methylated in their promoter regions not only in OSCCs but also in OLs. The number of methylated genes was significantly associated with the presence of dysplasia in OLs, which is known to be associated with a high risk of malignant transformation into OSCCs [7, 8]. This result indicates that accumulation of aberrant methylation might be associated with the malignant transformation of OLs, Aberrant promoter methylation is known to accumulate also in other organs, in high-risk tissues such as gastric mucosae with *Helicobacter pylori* infection, in liver tissue at the precancerous stage, in colonic mucosae with ulcerative colitis, and in esophageal mucosae [15, 16, 34–38]. These previous reports support the hypothesis that the accumulation of aberrant methylation in OLs produces epigenetic field defects leading to malignant transformation.

In addition to the methylation of multiple genes, the methylation silencing of a specific gene may be functionally involved in malignant transformation. The five genes methylation-silenced in OLs (*TSPYL5*, *CMTM3, NKX2–3, CLDN11*, and *RBP4*) were expressed in normal oral mucosae, which indicates the functional importance of these genes [34]. Especially, *TSPYL5* was most frequently methylated in OLs and was associated with differentiation levels of OSCCs (*p* < 0.01). The methylation silencing of *TSPYL5* has been reported in esophageal cancers, gastric cancers and malignant gliomas [33, 39, 40], and *TSPYL5* has been suggested to be a tumor suppressor gene [40]. Furthermore, *TSPYL5* is located on chromosome 8q22, which shows loss of heterozygosity (LOH) in OSCCs with high frequency [41].

In histopathological diagnosis, the presence of dysplasia, remains the golden standard for predicting the risk of cancerization in oral premalignant lesions [42]. However, this invasive approach cannot be repeated frequently because of its poor acceptance by patients. Furthermore, a diagnosis of dysplasia is also subject to the experience of a pathologist, and the consensus among pathologists is still poor [43]. On the other hand, quantitative DNA methylation analysis is currently available and is considered to be objective. Moreover, sufficient numbers of cells for methylation analysis can be obtained by non-invasive procedures [44], such as scraping of the oral mucosae. Thus, the risk characterization using aberrant DNA methylation in patients with OLs is considered clinically feasible. Accordingly, the accumulation of identified aberrant methylation is potentially useful as a risk marker of malignant transformation from OLs to OSCCs.

We identified a novel promoter methylation associated with the risk of malignant transformation of OLs. However, the number of OL samples was limited here. Therefore, it is necessary to validate the association by another larger population.

## Conclusions

Here, we identified aberrant promoter methylation of multiple genes in high-risk OLs. This result demonstrates that the accumulation of aberrant methylation in oral premalignant lesions produces an epigenetic field of cancerization.

## Abbreviations

5-aza-dC, 5-aza-2′-deoxycytidine; CGI, CpG island; LOH, loss of heterozygosity; MeDIP, methylated DNA immunoprecipitation; MSP, methylation-specific PCR; OL, oral leukoplakia; OSCC, oral squamous cell carcinoma; QOL, quality of life; TE, Tris-EDTA; UICC, Union for International Cancer Control; WHO, World Health Organisation.

## Additional files

Additional file 1: Table S1. List of primers for MSP. (XLSX 37 kb) Additional file 2: Table S2. Clinicopathological information of OL and OSCC cases. (XLSB 33 kb)
